# Effect of Marxist ideological and political education on students’ anxiety in colleges and universities

**DOI:** 10.3389/fpsyg.2022.997519

**Published:** 2022-09-20

**Authors:** Lingxia Guo

**Affiliations:** School of Marxism, North University of China, Taiyuan, China

**Keywords:** anxiety psychology, college Marxist ideological and political education, relieve effects, interpersonal relationships, metal health

## Abstract

With the rapid development of China’s economy, politics and culture, the frequency of college students’ anxiety has generally increased. Ideological and political educators in colleges should help college students to relieve anxiety and pressure in a timely manner, and constantly put forward effective and targeted methods. This paper analyzed the reasons for the anxiety of college students from three aspects: the way of dealing with the interpersonal relationship of college students, the degree of emphasis on college students’ academic performance, and the way of college students’ self-decompression. This paper proposed ways to strengthen the ability of thinking, carry out setback education, and improve the psychological quality of college students to realize the Marxist ideological and political education in colleges. This paper mainly used questionnaires and in-depth interviews to analyze the influence of mental health education courses on college students’ anxiety management ability. Among them, after the end of the mental health education course, a questionnaire was distributed to the selected research subjects to understand their stress management ability status after the course. Through the analysis and comparison of the results of the two questionnaires before and after, as well as the comparison of the conventional teaching methods of college students’ mental health education different from the teaching methods of group psychological counseling, it is necessary to understand the way college students’ deal with anxiety. The study found that 19.23% of the students had great anxiety about interpersonal communication problems, and 9.62% of the students reported that they had no major anxiety problems. Therefore, Marxist ideological and political education in colleges has become an important means for college students to vent their emotions, relieve psychological pressure and avoid adverse consequences.

## Introduction

Influenced by the test-oriented educational environment, scores have become the yardstick for parents and teachers to measure students. The one-sided education model that only emphasizes the score, not the practicality of knowledge, only the result and not the process, deviates from the ultimate goal of education. Exams replace the opportunity to become “people,” and students become the machines of examinations, lacking the ability of independent thinking and innovation. Even if the students break through the overlapping peaks and squeeze through the “single-plank bridge,” they enter the university with joy. However, in the face of more complex university life, students often show incompatibility, panic and anxiety in the face of failure, inability to persevere in the face of competition, and do not know how to deal with interpersonal relationships. The task of ideological and political education for college students is to cultivate students to form ideological viewpoints, political concepts and moral norms that meet social requirements, and then externalize them into corresponding behavior habits. Therefore, the ideological and political education of college students is helpful to relieve the anxiety of students.

Anxiety, tension and other negative emotions are not conducive to their own development. Chapman et al. (2017) has argued that people are unnecessarily anxious about perceived risk. Fryers et al. (2018) has argued that until recently, the social class distribution of common psychiatric disorders, mainly anxiety and/or depression, has been questioned. Kim and Koh (2018) has studied the structural relationship between avoidance of puppy love, self-esteem, anxiety, and smartphone addiction in college students. Zeeman et al. (2018) has suggested that implementing a learner-centered approach may improve students’ perceptions of readiness for learning and reduce anxiety about the course. Jun and Lee (2017) has argued that self-resilience has improved problem-solving ability and has reduced the effect of anxiety on problem-solving ability. However, the anxiety they put forward did not provide a good solution. This article has introduced Marxist ideological and political education in colleges and universities to optimize it.

The development of Marxist ideological and political education in colleges and universities is conducive to promoting the mental health of students. The purpose of the Oviedo U D study was to develop and validate a self-report tool for measuring learning in the cognitive, affective and psychomotor domains (Oviedo, 2019). Mcnamara K H has argued that higher education institutions are striving to educate students about environmental issues and support the development of sustainable innovation (Mcnamara, 2017). Thai N has investigated the different effects of flipped classroom and blended learning on learning performance, self-efficacy beliefs, and intrinsic motivation (Thai et al., 2017). Cooke has argued that traditional teaching methods have already employed within universities, including lectures and seminars, are beginning to come under scrutiny (Cooke, 2017). Lee K has argued that the rapid growth of online higher education in terms of course offerings and student enrollment is welcome (Lee, 2017). However, the effect of Marxist ideological and political education in colleges and universities they put forward is not obvious. This article has analyzed the concepts of anxiety, anxiety sources and anxiety environments by many scholars. Under the guidance of Marxist theory and psychology related theories, the influence of anxiety and anxiety environment on college students has been studied, and the relationship between anxiety and anxiety environment is interdependent and interactive. Secondly, anxiety has always been a problem in the field of psychological research. From the perspective of ideological and political education, this paper has studied the psychological anxiety of college students, and at the same time, has used ideological and political education to help college students put forward effective countermeasures to solve these psychological problems. Research has found that 24.36% of students are not confident in their appearance and have inferiority complex; 23.72% of students have reported that love problems have affected their self-growth. In addition, 41.67% of the students had academic problems such as being unable to keep up with their studies and relying on self-consciousness.

## Relief methods for students’ anxiety

### Anxiety

**FIGURE 1:**
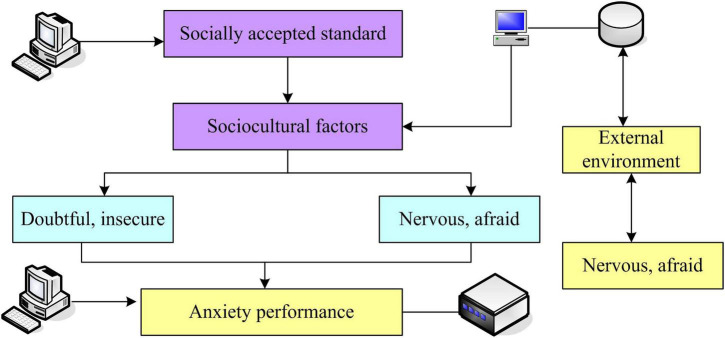
Factors influencing anxiety.

Anxiety is gradually formed on the basis of the individual’s birth with the accumulation of early experience, and will continue to develop and change with the various situations and experiences that the individual faces now and in the future. Therefore, various experiences in individual growth, especially the early experiences in the process of personal growth, such as family factors, interpersonal factors, life event factors, etc., will have a profound impact on the formation of individual anxiety. The stimulation factors, cultural factors and cognitive factors will also have a certain degree of influence on the formation of college students’ anxiety. This article is based on the premise of such theoretical assumptions to compile the questionnaire. Using the above point of view for anxiety, it is found that it is not the event itself that causes anxiety. It is the schema or thinking mode formed by people’s early experience, that is, cognition, that causes people’s anxiety about the event, and it is people’s negative automatic thinking about the event that causes anxiety. The stimulus situations that trigger anxiety vary from person to person, and specific stimulus situations will activate specific schemas or thinking patterns, that is, cognition. As a result, the stimulus situation is perceived by negative automatic thinking as dangerous. The individual develops a sense of insecurity, which leads to manifestations and symptoms of anxiety. And it induces “safety behaviors” to escape anxiety, which in turn strengthen cognitive structures and simultaneously reinforce the manifestations and symptoms of anxiety (Baldwin et al., 2017). The influencing factors of anxiety are shown in Figure 1.

**FIGURE 2:**
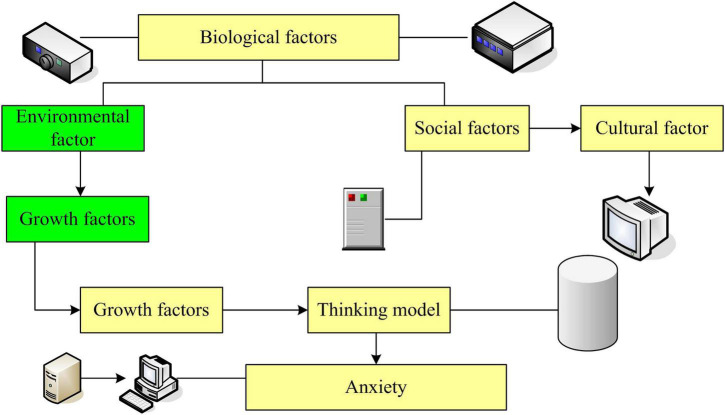
Comprehensive conclusion model of the influencing factors of college students’ anxiety.

Through the above theoretical analysis, we can see that there are many factors that affect anxiety, including biological factors, living environment factors, stimulating situational factors, cultural factors, cognitive factors and so on. They each affect the anxiety of college students in different ways. The comprehensive conclusion model of the influencing factors of college students’ anxiety is shown in Figure 2. There are two main lines in this model: one of the main lines is that individuals have individual differences (such as the intensity, balance and flexibility of higher neural activity) in biological factors from birth. From birth, individuals have different sensitivities and perception thresholds, so that the arousal systems to stimuli are different. But this kind of arousal system is not static. With the growth of the individual, through the joint action of environmental factors and social factors, the role of biological factors is increased or weakened by subsequent experiences. Thus, different arousal systems to stimuli are formed after individual growth, which is the biological basis of individual’s response to stimuli. The second main line is that under the condition of the unique biological basis of the individual at birth, through the combined action of environmental factors and social factors in the process of individual growth, it undergoes continuous assimilation and adaptation. A thinking mode with a certain tendency is formed, that is, a cognitive mode or schema, which is the cognitive basis of the stimulus response. When there is a stimulating situation that causes anxiety, it acts on the individual’s biological arousal system and cognitive mode. It makes the individual feel the anxiety emotionally and triggers the biological arousal system of the anxiety, so that the performance and behavior of the anxiety will appear. From this model, it can be seen that it is not the stimulation situation itself that directly produces anxiety, but the stimulation situation acts on the individual’s biological arousal system and cognitive mode. Under the combined action of biological arousal system and cognitive mode, the reaction and expression of anxiety can be produced in the stimulus situation. The cognitive model is closely related to the long experience of biological arousal, the social and cultural environment and its biological basis. It can be seen that among the various anxiety emotions such as study anxiety, exam anxiety, social anxiety, and employment and career choice anxiety in college students, it is by no means caused by a single factor stimulating situation. Rather, it is the result of the combined action of biological factors, environmental factors, social and cultural factors, and stimulating situations, and is the result of the combined precipitation of these factors over time (Hitchcock et al., 2017).

### Marxist ideological and political education in colleges and universities

#### Strengthen the ability to think

It is used in various ways and in various occasions to cultivate the dialectical thinking ability of college students. In addition to focusing on cultivating students’ critical thinking ability in ideological and political theory courses and other philosophy and social science courses in colleges and universities, the cultivation of students’ dialectical thinking should also be strengthened in the teaching of science and engineering in colleges and universities. In various party and group activities and various ideological and political education activities in colleges and universities, especially in various appraisals, selections, competitions, commendation activities, in various student assistance, difficulty solving, criticism and punishment work, the students have been strengthened to cultivate their thinking ability. It enables students to see themselves correctly, to see others correctly, to face progress and achievements correctly, and to face setbacks and mistakes correctly. Students are taught to analyze the pressure problems they encounter with a dialectical attitude at all times. Students are educated to give full play to the role of dialectical thinking, and to understand things in a developing way of thinking and thinking. Educating students to look at stress from a developmental perspective, students’ ability to see things dialectically should be cultivated, so that they can see both sides of things. Only when students have the thinking ability of dialectically looking at problems, can they better cope with anxiety problems, and they will not feel helpless when viewing, analyzing and solving anxiety (Prenoveau et al., 2017).

#### Carry out setback education

The ability of college students to deal with setbacks is enhanced, which effectively promotes the all-round development of college students. This article has mainly summarized from the aspects of clarifying the orientation of the mental health education course, enriching the content of the mental health education course, expanding the teaching methods of the mental health education course, and strengthening the teaching management of the mental health education course. The prediction of the previous stage is concatenated with the original anxiety facial image features, and then the confidence and affinity of the anxiety relief test results of the next stage are predicted in a more refined manner. The iterative process (Kishita and Laidlaw, 2017):


(1) A=β⁢(O,A,B)

Among them: O, A are the modifiable internal parameters of the model, which are used to adjust the iterative effect of confidence and facial feature affinity.

And the deviation between the anxiety facial feature encoding of the image to be tested and the existing anxiety facial feature encoding in the face information database is calculated (Delgadillo et al., 2017):


(2) $$\beta =\sqrt{{\left({\text{x}}_{\text{j}}-{\text{x}}_{\text{i}}\right)}^{2}+{\left({\text{x}}_{\text{j}-1}-{\text{x}}_{\text{i}-1}\right)}^{2}+\cdots \,{\left({\text{x}}_{0\,\text{j}}-{\text{x}}_{0\,\text{i}}\right)}^{2}}$$

Among them: j is the anxiety face key point value extracted by Dlib.

Further reasoning about SVM: The goal of SVM is to iteratively update the parameters of the test study sample, and at the same time, the minimum distance between the scores before and after the class is calculated and the geometric difference criterion is calculated (Pelgrum, 2017):


(3) $$\text{y}\,\left(\frac{\text{w}}{||w||}+\frac{\text{b}}{||w||}\right)\,\text{x}\geq \gamma$$

#### Improve the psychological quality of college students

College students are people who are engaged in certain activities under certain social relations, and their ideological progress and development cannot be separated from the adaptation to the society, especially the adaptation to the economic foundation. The psychological quality of college students is improved, and a harmonious psychological environment is created for accepting ideological and political education. Society is composed of people, and people’s own health is the foundation and premise of social development. An important sign of social progress is the concern for people’s own health. The complex reality of the social transition period impacts the psychology of college students. It leads to psychological imbalance, intensified psychological confusion, and unhealthy emotions such as depression and anxiety. In the National Conference on Strengthening and Improving the Ideological and Political Education of College Students, the problem of students’ mental health education has been mentioned many times. Psychological counseling takes the psychology of college students as the starting point. The purpose is to eliminate the psychological distress of the students and create a good psychological condition for solving the ideological, political and moral problems of the students. Therefore, the introduction of psychological counseling into the practice of ideological and political education of college students is conducive to improving the psychological quality of students.

In this paper, three empty arrays are established to count the action characteristics of anxiety degree 0, anxiety degree 1, and anxiety degree 2. Finally, the time information of different anxiety levels is saved in the corresponding array. The time proportions B0, B1, B2 of anxiety level 0, anxiety level 1, anxiety level 2, and anxiety level 3, and the frequency of anxiety fluctuations per unit time are calculated (Costa et al., 2017).


(4) $$B_{0}=C_{0}/\left(C_{0}+C_{1}+C_{2}\right)$$

(5) $$B_{1}=C_{1}/\left(C_{0}+C_{1}+C_{2}\right)$$

(6) $$B_{2}=C_{2}/\left(C_{0}+C_{1}+C_{2}\right)$$

Finally, according to the anxiety distribution obtained by the analysis, the report is automatically compiled and the scale is output. The identity of the subjects, the analysis time period, the duration of different anxiety levels and the proportion of the total duration, the frequency of occurrence of different anxiety levels, and the final anxiety level evaluation score were counted and suggestions were given.

Taking the offline anxiety evaluation results of multiple evaluators as the real score, the evaluation of action emotion analysis in speech situations is practical and robust (Tuapawa, 2017).


(7) $$Z_{Q}=\left|\frac{X_{Q}-X_{T}}{X_{Q}}\right|$$

*X*_Q_ is offline score, *X*_T_ is system score.

#### Anxiety psychological relief assessment

This paper involves students’ stress performance, stress sources and stress analysis of college students. The data comes from two aspects: self-made questionnaires, distributed data collected and analyzed, and the results of investigation and census provided by a psychological counseling center in a university. The questionnaire is based on the “Chinese College Students’ Mental Health Assessment System.” The questionnaire design is universal and pertinent by means of self-made questionnaires. It draws on the opinions and suggestions of mental health experts who have a certain representativeness in mental health in a psychological education center of a university, and psychological counseling teachers working on the front line. The 25 selected questionnaire questions were produced. The results of the annual psychological investigation of college students in a university and the data analysis in the analysis provide authoritative support for the study of college students’ stress problems (Anderson and Collis, 2017).

1. (1) Overview of the questionnaire survey

The questionnaire adopts a self-made stress questionnaire. This survey adopts the method of random sampling, with a university as the main investigation point, with the help of the classroom of ideological and political theory teachers. There were 600 respondents, including 300 boys, 300 girls, 150 freshmen, 150 sophomores, 150 juniors, and 150 seniors.

(2) Data processing situation

This article analyzes the current situation of college students’ psychological stress, and the data involved mainly include: First, the questionnaires are compiled according to the “Chinese College Students’ Mental Health Evaluation System” research group of the Ministry of Education. It adopts self-made questionnaires and uses the WeChat platform to analyze the data collected with the help of the questionnaire network. With the help of teachers from the School of Marxism, this questionnaire is a method in which the teachers issue the QR code of the questionnaire in the classroom, and students scan the QR code to answer the questions. And teachers publish questionnaires to the WeChat group of the public course of Marxist theory, and students click on the questionnaire to answer the questions, complete the questionnaire, and conduct data analysis on the data through the questionnaire network (Mayorova et al., 2018).

Second, because a university conducts a psychological census and investigation for students every year, this part of the data is more authoritative, so the author adopts part of the content of the psychological census and investigation to study and analyze the stress performance of students and the factors that cause stress. In view of the confidentiality of students’ personal information, no student information will be disclosed here, but only a part of the psychological census and investigation data of a university is used to summarize and analyze the psychological pressure of college students, and will not disclose any information of students.

This paper adopts the method of random sampling. In university classrooms, group testing is carried out with the help of self-study time, and all subjects are required to participate voluntarily. Before the implementation of the test, the tester indicated the content and purpose of the test, which took about 30 min, and all the questionnaires were collected on the spot. The recovered valid questionnaires were input into the computer uniformly, and SPSS13.5 statistical analysis software (SPSS is a general term for software products and related services used for statistical analysis operations, data mining, predictive analysis, and decision support tasks.) was used for statistical analysis.

Let X,*X2*, …..*Xn* denote the overall test student sample, X denote the mean value of the test student sample, and the central moment of the test student sample is (Sierra et al., 2017):


(8) $$\text{M}=\frac{1}{\text{N}}\,\sum {\left({\text{X}}_{\text{j}}-\bar{\text{X}}\right)}^{2}$$

*m* represents the central moment of the *i*-order test student sample, the skewness Z_F_ and the kurtosis F_D_ of the normal distribution are both 0, and the skewness and kurtosis are defined as (Rani1 and Widyowati, 2021):


(9) $${\text{B}}_{\text{X}}=\frac{{\left({\text{X}}_{\text{j}}-\bar{\text{X}}\right)}^{2}}{{\text{Z}}_{\text{F}}}+\frac{\text{E}\,{\left({\text{X}}_{\text{j}}-\bar{\text{X}}\right)}^{4}}{{\text{F}}_{\text{D}}}$$

T-test: Establish hypotheses and determine the significance test level set to 0.05. The test statistic is calculated for the two-test Student-Sample *t*-test as (Alyouzbaky et al., 2021):


(10) $$\text{T}=\frac{\bar{\text{X}}-\bar{\text{Y}}}{\sqrt{\text{M}+\text{N}}}+\frac{{\left({\text{S}}_{\text{j}}-\bar{\text{S}}\right)}^{2}}{\text{M}+\text{N}}$$

Among them, M and N are the sample size of the test students and the standard deviation of the test students’ samples.

## Psychological relief effect of students’ anxiety

Under the psychological pressure of college students will produce tension, anxiety, and then do harm to their health. In the daily life and learning process of college students, changes in thoughts and emotions often occur due to changes in life, study and interpersonal relationships, which in turn affect their normal life and study, and even affect their physical and mental health. College students are helped to find the right way to express their emotions and relieve their stress. The role of psychological pressure and its possible consequences are correctly recognized, so that college students are psychologically prepared for possible excessive psychological pressure, and actively learn how to vent their emotions and relieve pressure, such as formulating practical learning and life goals, cultivating interests and hobbies, strengthening physical exercise, establishing and expanding good interpersonal relationships, etc. (AlQAheri, 2021).

There was a statistically significant difference in the scores of SAS (SAS is the Anxiety Self-Assessment Scale.) and HAMD between the self-differentiation groups, and the level of self-differentiation was negatively correlated with anxiety and depression levels, which were further grouped and compared. The higher the level of self-differentiation, the lower the level of anxiety and depression. The self-differentiation scores at different levels are shown in Table 1.

**TABLE 1:** Self-differentiation scores at different levels.

Degree of self-differentiation	Anxiety (score)	Depression (score)
Low	0	0
Preliminary	50	20
Middle	40	16
High	35	10

**FIGURE 3:**
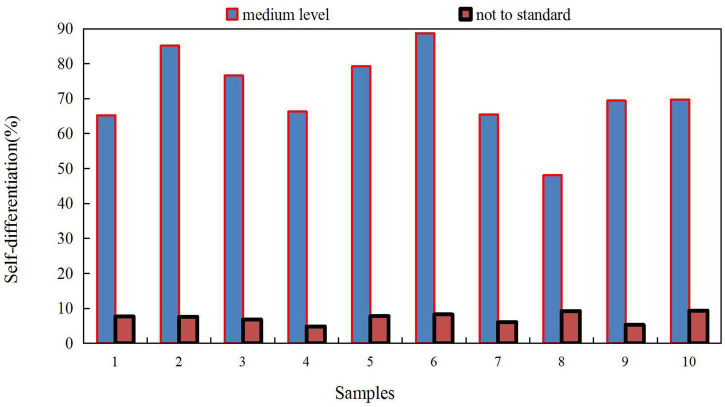
Basic situation of college students’ self-differentiation.

College students have a high level of self-differentiation, and college students above the middle level account for 89% of the total, indicating that most college students can maintain a balance between reason and emotion, and can maintain calm in the face of pressure. But there are still 11% of college students’ self-differentiation level not up to the standard. Such students are easily confused by trivial matters, and are prone to mood swings. There are two ways in their life: one is to reduce stress by approaching and relying on others; the other is to avoid others to reduce anxiety. This result also shows that a considerable number of college students still lack the skills to regulate emotions, deal with interpersonal relationships, deal with emergencies, and adapt to the new environment. This requires our high attention. Therefore, teachers, especially those engaged in ideological and psychological education, should do a good job in guiding and educating, so as to improve college students’ autonomy and ability to deal with things without emotion. They are made to deal with difficulties and setbacks with equanimity, and thus to deal with problems according to their own strength. This can not only enhance the mental health of college students, but also promote their development of healthy personality. The basic situation of college students’ self-differentiation is shown in Figure 3. The divergence between the concept of family education and the training goal of ideological and political education for college students is an important factor restricting the development of ideological and political education for college students. Therefore, parents should pay attention to changing the traditional utilitarian educational concept and avoid “only fractional theory,” which only depends on the academic performance and not on the comprehensive and healthy development of personality and morality. Parents should also cultivate their children’s sense of social responsibility and the all-round development of morality, intelligence, body, beauty and labor. Parents should also recognize the adverse effects of authoritarian or laissez-faire education methods on children, adopt democratic and reasonable education methods, enhance the democratic atmosphere of the family, and cultivate children’s healthy personality and positive moral quality. Parents should also improve children’s autonomy and initiative in learning and life, guide children to change the external motivation of learning into internal motivation, increase children’s self-confidence in learning, and improve self-efficacy. The formation of self-efficacy is beneficial to college students’ initiative and enthusiasm for self-education and self-development in future study, life and work. At the same time, parents should also improve their own ideological and moral qualities, and set good examples for their children.

Schools should set up corresponding psychological counseling institutions, such as emotional catharsis rooms or life guidance rooms, to provide hardware facilities for students to carry out individual psychological counseling to ensure the effective development of psychological counseling. Schools and colleges can organize students to listen to lectures, discussions and discussions, and set up special psychological counseling institutions. Corresponding psychological counseling work is carried out for students of different grades to provide students with professional psychological guidance. The head teacher can organize students to conduct group discussions to expand the realization form of psychological counseling. For example, for freshmen, it is necessary to do a good job in the education of students and help them quickly change their roles to adapt to university life. Leaders of schools and colleges or head teachers and counselors of various grades must not despise this work, formalize them, and complete the task in a fancy way. In the form of lectures or class meetings, professional teachers should be equipped to appropriately introduce the characteristics of university study, professional conditions, and interpersonal characteristics. For senior students, the focus of psychological counseling can be placed on practical issues such as further education and employment, and social experts are invited to “show up.” Students are given vocational guidance to help students improve their professional quality and ability by combining their own and social needs.

In order to establish a more certain relationship between self-differentiation, anxiety and depression, this paper conducted a two-step multiple regression analysis. The student’s depression score was used as the dependent variable. The four subscales of DSI (DSI is a depression status questionnaire.) were used as the first-level predictors, and the subjects’ anxiety scores were used as predictors. In regression analysis, the variance inflation factor of each variable is between 1.00 and 1.38, indicating that there is no serious multicollinearity in the data. In the second layer, the three factors of self-differentiation-emotional response, emotional disconnection and integration with others have significant negative predictive effects on depression, while self-position has no predictive effect on depression. And in the second layer, anxiety scores have a positive predictive effect on depression. After entering the factors from the first layer to the second layer, the prediction ability of the whole equation increases from 22 to 45%, an increase of 23%. The ego position value decreased, and the significance level decreased. It can be seen that self-position has no significant predictive effect on depression. Anxiety partially mediates between self-differentiation and depression. The multiple regression results of self-differentiation, anxiety and depression are shown in Table 2.

**TABLE 2:** Multiple regression results of self-differentiation, anxiety and depression.

Step	Variable	Variance inflation factor
Level one	Emotional response	0.2
	Ego position	0.15
Level two	Emotional severance	0.3
	Integrate with people	0.35

Ideological and political educators in colleges and universities should strengthen contact with school psychological counselors. When it is found that some students may have psychological barriers, it is necessary to ensure the smooth flow of communication channels, get in touch with psychological counseling personnel in time, and transfer students to professional psychological counseling institutions for treatment to avoid unnecessary consequences. Psychological counseling institutions in colleges and universities should also actively cooperate with the work of ideological and political educators. The two forces exert their respective advantages and form a joint force to jointly promote the application of psychological counseling in the ideological and political education of college students. In addition, the Internet plays an important role in the current study and life of college students. Colleges and universities can also develop online psychological counseling platforms to provide real-time guidance and help for college students’ emotional communication and emotional venting. The self-differentiation level of male and female college students only has gender differences in the perspective of emotional response, and there is no gender difference in other levels. There are obvious differences in the self-differentiation levels of male and female college students in the dimensions of self-position, emotional reactivity and getting along with others.

**FIGURE 4:**
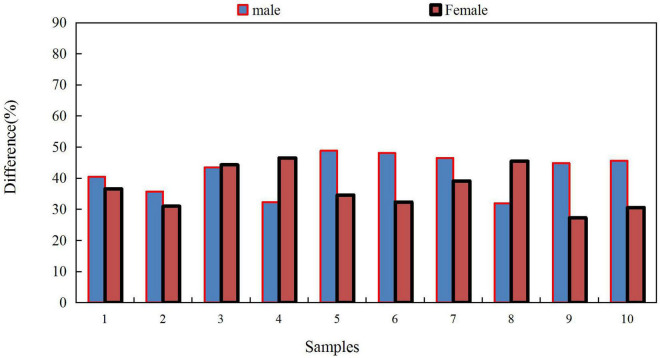
Differences in the performance of male and female college students.

In terms of gender, there is no obvious difference in the self-differentiation of college students, but there are some differences in self-position. The traditional Chinese concept believes that men are rational, brave, and active. In the process of childhood training, boys pay more attention to the cultivation of reason. In comparison, men are more likely to judge their position rationally and correctly. However, further research is needed in view of the controversial findings on the effect of gender differences on the self-differentiation of college students. The difference between male and female college students is shown in Figure 4.

**FIGURE 5:**
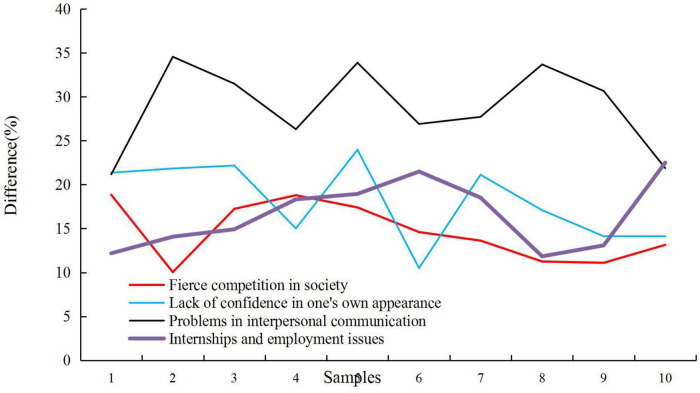
Influencing factors of student anxiety.

The main anxiety and psychological problems of college students are in the following aspects: 60.26% of the students believe that the fierce competition in the society is the source of stress. 44.87% of the students think that they are under great pressure in terms of tuition fees, living expenses and other economic issues. 41.67% of the students have great anxiety about academic problems such as being unable to keep up with their studies and relying on self-consciousness. 28.85% of the students report that their parents and relatives bring anxiety about their discipline and high expectations. 24.36% of the students are not confident about their personal appearance, and have low self-esteem. 23.72% of the students report that love problems affect their self-growth to a large extent. 19.87% of the students believe that anxiety is obvious in employment and internship. 19.23% of the students have greater anxiety about interpersonal communication problems. 9.62% of the students report that they do not have much anxiety. Anxiety is actually the driving force for progress. This part of the students has better cognition of anxiety and the thinking way of thinking to deal with anxiety. The sources of students’ anxiety are mainly manifested in four aspects: social anxiety psychological source, school anxiety psychological source, family anxiety psychological source and students’ personal anxiety psychological source. Only by figuring out where students’ anxiety comes from, can students be helped to solve anxiety problems and provide more targeted measures. Among them, the main aspect of the contradiction is the internal ideological contradiction of college students. The formation and development of college students’ ideological and moral character are the result of the comprehensive effect of their psychology, thought, behavior and environment. The influencing factors of student anxiety are shown in Figure 5.

**FIGURE 6:**
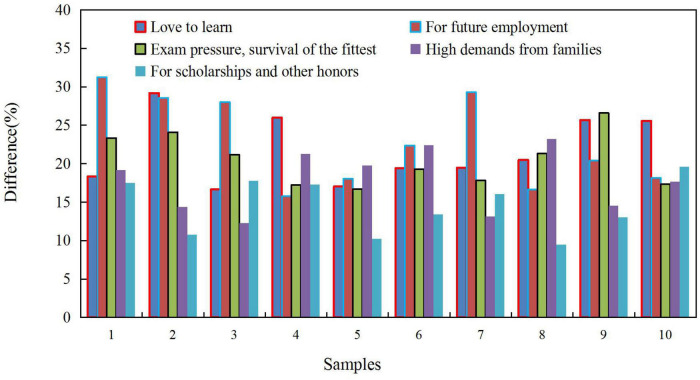
Students’ learning purpose and motivation.

The students’ learning purpose and motivation are shown in Figure 6, and 73.42% of students believe that future employment is the motivation for learning. The pressure of academic examinations is the driving force for 55.7% of students. 31.01% believe that the motivation for learning comes from the high demands given by the family. 18.35% of students believe that study is for honors such as scholarships. It even reflects that 5.06% of the students are not motivated to study, and 64.56% of the students in the latter three are under pressure to study. Due to various factors, they show stress, anxiety and worry about their academic performance.

By understanding the purpose and motivation of students’ learning, the stress performance of students with serious psychological problems reflected in the data will be more clear and definite. According to the results and analysis of the psychological investigation of undergraduates in the 2017—2018 academic year provided by the psychological counseling center of colleges and universities, it can be seen that the degree of emphasis placed on academic performance by students with serious psychological problems affects the changes in the students’ psychological pressure. Student A has a lot of pressure to review the postgraduate entrance examination, pays too much attention to the postgraduate entrance examination, and regards the postgraduate entrance examination as a benchmark for success or failure in life, resulting in insomnia, frequent irritability, and mild depression. Student B has a lot of academic pressure and fails the monthly exams many times. In fact, the student attaches great importance to each exam and puts in high effort, but his grades are not ideal. Student C, who was originally an international student, has been unable to adapt to the state of university study after enrolling. He often skips classes, sleeps in the dormitory or plays with his mobile phone, and participates in less extracurricular activities. In the end, it leads to many failed subjects, the less the grades reach their ideal state, the more they hate studying; the students who fail the D test in many subjects are more likely to drop out of school. Student D fails the multi-subject exam, may drop out of school, is withdrawn, and is a loner. He is addicted to novels and movies and can’t stop to study. Student E is mildly disgusted with learning, focuses on entrepreneurship, has no interest in any course, and pays less attention to learning; Student F fails more subjects, has a poor sense of professional identity, and is completely not interested with his own major. He didn’t pay attention to his academic performance and kept his grades; student G was a liberal arts student, but because of family pressure, he chose to study science, and he had inner resistance. After entering the university, he was resistant to mathematics-related majors and was not strict with himself. He received an academic warning in the first year of his sophomore year, and at the beginning of the second year of his sophomore year, he got better, but he developed depression during the final exam and did not take the final exam. The commonality of the above students is shown in two aspects: one is that they pay too much attention to learning. They demand too much of themselves in their studies. Often, the more effort students put in, the more anxious they become. However, students with unsatisfactory grades, or even failing grades, become more anxious, insomniac, irritable, and emotional. One is the state of not paying attention to learning, or even being completely tired of learning. Students find that their professional knowledge runs counter to their expectations, perceptions and interests. The main pressure is truancy, avoidance of learning, weariness of learning, and severe depression tendency. It can be seen from this that it is very necessary for colleges and universities to carry out ideological and political courses to relieve anxiety.

**FIGURE 7:**
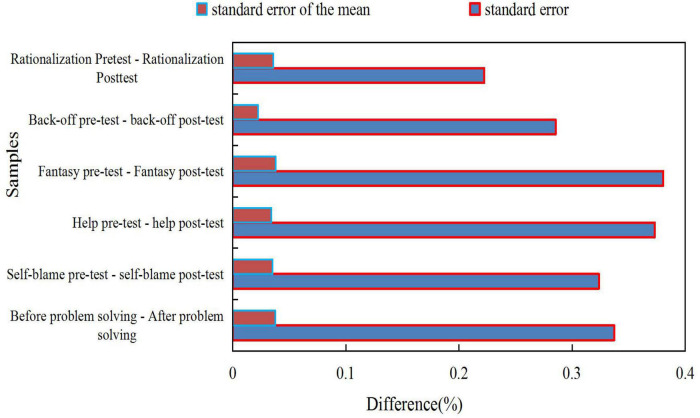
*T*-test results.

After the Marxist mental health education course was taught, the *t*-test (*t*-test is mainly used for normal distribution with small sample size and unknown population standard deviation.) Sig value of the problem-solving factor was 0.016, which was less than 0.05, that is, there was a significant difference in the problem-solving factor. In the *t*-test of the self-blame factor, the Sig value is 0.905, which is greater than 0.05, and there is no significant difference in the self-blame factor. Similarly, there is no significant difference in other factors of coping style. After the Marxist mental health education course was taught, the average score of the problem solving factor of college students increased from 0.770 to 0.800. After the Marxist mental health education course was taught, the average score of the problem solving factor of college students increased. It shows that college students are more active in coping with stress after the Marxist mental health education course is taught, indicating that the Marxist mental health education course is helpful to improve the anxiety management ability of college students. The *T*-test results are shown in Figure 7.

**FIGURE 8:**
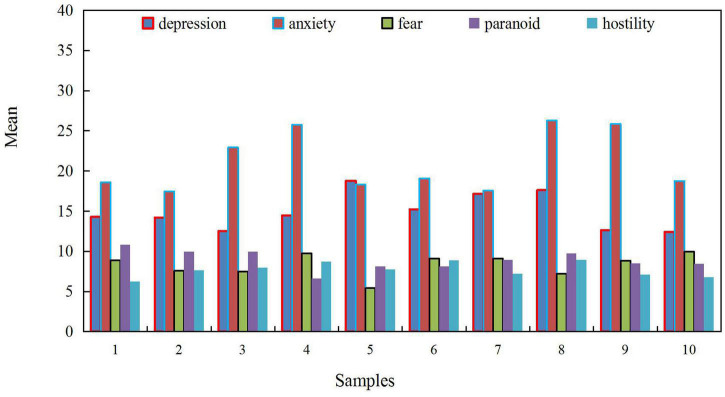
The influence of anxiety level of college students after the course of mental health education.

The average overall score of college students after the Marxist mental health education course is 154.141. Before the class, the average total score of the college students on somatization and other factors is 154.838. The score has declined, indicating that the overall mental health level of college students has risen after the Marxist mental health education course was taught. The results are consistent with those of the in-depth interviews. In interviews with college students, some classmates said that by taking Marxist mental health education courses, especially the content of anxiety and mental health, after correctly understanding the concept of anxiety, the source of anxiety, the reaction of anxiety and the common anxiety of college students, and learning the strategies of coping with anxiety, they obviously feel that their anxiety is lessened than before. A college freshman said: By determining a goal that suits him, making a plan and arranging life tasks reasonably according to the plan, exercising more after study, participating in extracurricular practical activities, combining work and rest, and learning to actively relax, when encountered setbacks, my classmates, brothers and sisters and teachers helped. The discomfort and anxiety before have been swept away, and now the university life has been basically adapted. Another student also said after the class that he has gained a lot through the group psychological counseling class “Walking with Anxiety.” It not only helps others to solve problems, but also has a sense of accomplishment, and also gets the methods given by others, solves problems, reduces a lot of anxiety, makes life smoother, and feels warm in the world. Figure 8 shows the influence of college students’ anxiety level after the Marxist mental health education course is taught.

## Conclusion

Combining the research on the pressure of college students in colleges and universities, it can be found that in recent years, the Marxist ideological and political education in colleges and universities has played an important role and achieved good results, such as, the continuous improvement of college students’ psychological bearing capacity, the continuous optimization of students’ psychological quality, the continuous reduction and prevention of the occurrence of students’ psychological diseases, and the continuous promotion of students’ comprehensive, harmonious and healthy development and so on. Marxist ideological and political educators in colleges and universities reflect on the achievements and deficiencies of Marxist ideological and political work on the basis of “environmental education theory” and “human nature theory.” It is an inescapable responsibility to take more effective and targeted measures to adjust and relieve the pressure of college students. Based on the current situation of college students’ pressure, this paper has discussed the pressure of college students in various environments such as social environment, educational environment, family and dormitory environment. Attribution analysis has been carried out, combining the subject knowledge of psychology with the subject knowledge of ideological and political education, combining theory with practice, and has put forward research thinking on the problems and countermeasures of ideological and political education for college students under the anxiety environment. Admittedly, there are still many shortcomings in the research work of this paper, and there are still many unsolved problems. For self-made questionnaires, there are still limitations that need to be further revised and improved in practice due to time, space and many other factors. Because the research on the stress environment is not comprehensive and authoritative enough, the coverage of the selected research samples is not comprehensive enough. It may bring some errors to the conclusions of the research. The exploration of the countermeasures to relieve the anxiety of college students under the environment of Marxist ideological and political education is also a little less in-depth and needs to be further explored in the future.

## Data availability statement

The original contributions presented in this study are included in the article/supplementary material, further inquiries can be directed to the corresponding author.

## Author contributions

The author confirms being the sole contributor of this work and has approved it for publication.
